# Evaluation of Osteogenic Potential of Fucoidan Containing Chitosan Hydrogel in the Treatment of Periodontal Intra-Bony Defects—A Randomized Clinical Trial

**DOI:** 10.3390/gels9070573

**Published:** 2023-07-13

**Authors:** Shruthi Eshwar, Kranti Konuganti, Supriya Manvi, Aarya N. Bharadwaj, Sudarshan Sajjan, Sateesha Shivally Boregowda, Vipin Jain

**Affiliations:** 1 KLE Society’s Institute of Dental Sciences, Bengaluru 560022, India; 2 Faculty of Dental Sciences, Ramaiah University of Applied Sciences, Bengaluru 560054, India; 3 Acharya & BM Reddy College of Pharmacy, Bengaluru 560090, India

**Keywords:** fucoidan-chitosan, injectable hydrogel, osteogenic potential, periodontal infra-bony defects, human experimental trial, physico-chemical characterization, biological characterization

## Abstract

Periodontal diseases significantly impact about half of the global population, and their treatment often encompasses relieving symptoms as well as regenerating the destroyed tissues. Revolutionary research in the management of periodontal disease includes biomaterials, a boon to re-generative dentistry owing to their excellent biological properties: non-toxicity, anti-inflammatory, biocompatibility, biodegradability, and adhesion. This study aimed to fabricate an injectable fucoidan containing chitosan hydrogel and prove its effectiveness in periodontal bone regeneration. The injectable hydrogel was prepared using the sol-gel method and was subjected to various physical, chemical, and biological characterizations to understand its efficacy in formation of new bone. The effectiveness of the developed hydrogel was assessed in periodontal bony defects to study the soft and hard tissue changes. A total of 40 periodontitis patients with bony defects were recruited and randomized into two groups to receive fucoidan-chitosan hydrogel and concentrated growth factor, respectively. Customized acrylic stents were used to guide the hydrogel placement into the defect site. Post-surgical changes in clinical parameters were assessed at 3, 6, and 9 months to appreciate the soft and hard tissue changes using repeated measures analysis of variance and Bonferroni’s post hoc test. Significance was kept at 5%. The porosity, water uptake of the prepared hydrogel showed good efficacy, with particle size of the fucoidan containing chitosan hydrogel of 6.000 nm. The MG-63 osteoblasts cell line revealed biocompatibility, biodegradability and showed slow and sustained drug release, increased cell proliferation, and enhanced alkaline phosphatase secretion. Mineralization assay was greatest in the fucoidan containing chitosan hydrogel. Clinically, it exhibited significantly lower probing depth values and a higher mean improvement in clinical attachment level as compared to the concentrated growth factor (CGF) group at the end of 3 and 6 months (*p* < 0.05). The mean of the defect fills in the fucoidan containing chitosan group was 1.20 at the end of 9 months (*p* < 0.001) as compared with defect fills observed in the CGF group. The presence of fucoidan in the hydrogel significantly contributed to bone regeneration in humans, thus strengthening its potential in tissue engineering. Fucoidan-chitosan will be a promising biomaterial for bone tissue regeneration.

## 1. Introduction

Bone is a dynamic connective tissue that protects internal organs, helps in locomotion and in the maintenance of homeostasis. A common finding in various systemic and dental disorders, bone disorders as a result of trauma are characterized by disturbed natural healing, resulting in functional and structural oddness [1]. Periodontitis is an infectious disease of the tooth-supporting structures affecting about 20–50% of the world’s population [2]. The rationale for periodontal treatment is bifold: to relieve symptoms as well as regenerate the destroyed tissues. Alternative regenerative procedures take the forefront when clinical periodontal therapy fails to regenerate lost periodontal tissues [3]. Current research and developments in dentistry have revolutionized the approach to managing periodontal disease [4]. While tissue engineering of periodontal structures is quite challenging due to its complex anatomy of soft tissue interspersed between two distinct hard tissue structures [5], an interplay among materials, cells, and bioactive signals could initiate regeneration. Different grafts, materials, barrier membranes, and bone substitutes can restore osseous defects [6]. Effective yet limited scope due to additional surgery, inadequate bone supply, inappropriate biodegradation, immune response, and low tissue compatibility has shifted the focus to the fabrication and convention of natural biomaterials [6].

Natural biomaterials and polymers have gained significant interest in the field of regeneration due to their superior biological properties. Preformed scaffolds or hydrogels, primary components of bone tissue engineering, have been widely researched and proved as promising bone regenerative materials. They are three-dimensional hydrophilic polymer chains which exhibit excellent mechanical strength, maximum penetration, and mimic extracellular matrix. However, certain limitations like poor integration, limited penetration, and cost have paved the way for the use of injectable hydrogels in bone regeneration [1].

Chitosan, a deacetylated chitin, is a natural polymer exhibiting exceptional biological properties like non-toxicity, anti-inflammatory, adaptability, biocompatibility, biodegradability, and bio-adhesion, thus stimulating osteoconductive functions and playing a pivotal role in tissue engineering, drug delivery, and wound healing [7]. Its main drawbacks like lack of bioactivity and degradation cross-link it with other polymers such as alginate, gelatin, hyaluronic acid, amylopectin, carbon nanotubes, poly (methyl methacrylate), polylactic acid, growth factors, and calcium phosphate to enhance mechanical and osteoconductive properties of chitosan [8]. However, cross-linked chitosan alone fails to improve osteogenic potential completely [9]. Hence, with this interest, a wider literature search revealed the application of fucoidan brown seaweed for biomedical purposes.

Fucoidan is a sulfated polysaccharide in brown seaweeds, like heparin, and characterized by most biological activities, including cell proliferation and differentiation. Recent research shows fucoidan’s biomedical applications, which include anti-inflammatory, antiviral, antibacterial, anti-coagulant, anti-obesity, and anticancer properties [10]. Fucoidan for bone tissue regeneration has been comprehensively reported, presenting both osteoclast and osteogenic potential. Fucoidan is known to promote bone markers confirmed by ALP activity, osteogenic gene expressions such as osteopontin, Runx2, and mineral deposition, and increase the proliferation of osteoblast MG-63 cells, human alveolar bone marrow-derived mesenchymal stem cells, and human amniotic fluid stem cells [11,12].

Fucoidan containing chitosan hydrogel is now being researched to study its combined properties and has shown superior wound healing in animals, with faster dermal papillary formation and the closure of the wound after 14 days of treatment [13]. Similarly, chitosan-alginate-fucoidan scaffold has been shown to be a promising material for bone regeneration [14]. Fucoidan-induced osteogenesis studied through cellular mechanisms shows activation of mitogen-activated protein kinases (MAPKs), including JNK and extracellular signal-related kinase (ERK), bone morphogenetic protein-2 (BMP-2), and Smad 1/5/8 signaling [12]. Currently, fucoidan encourages PI3K/Akt signaling pathways for osteoblastic differentiation in stem cells and angiogenesis during bone repair. In addition, various studies have been reported on the role of the Wnt/β catenin pathway in bone biology [15].

The extent of influence of fucoidan on the osteogenic potential of periodontal cells is unexplored and hence, we aimed to develop a fucoidan containing chitosan hydrogel, study its characteristics, and to compare and evaluate its osteogenic potential against concentrated growth factor in managing periodontal intra-bony defects.

## 2. Results and Discussion

Tissue engineering is an upcoming interdisciplinary field, which focuses on replacing lost tissues and organs. Regeneration encompasses the use of four approaches: cells, scaffolds, signaling molecules, and a genetic matrix. Since its inception, this scientific innovation has stimulated research on various materials used to accomplish bone regeneration. Ideal features like biocompatibility, biodegradability, ease of application, adaptability to the tissues, and bone repair are essential to successful regeneration. In this regard, natural polymers stand out and are researched globally, owing to their excellent biological properties, especially in the replacement of body parts. Thus, thrust areas in tissue engineering investigations are directed towards the repair of many different tissues like bone, cartilage, cornea, skin, liver, heart, and periodontal tissue.

**Figure 1:**
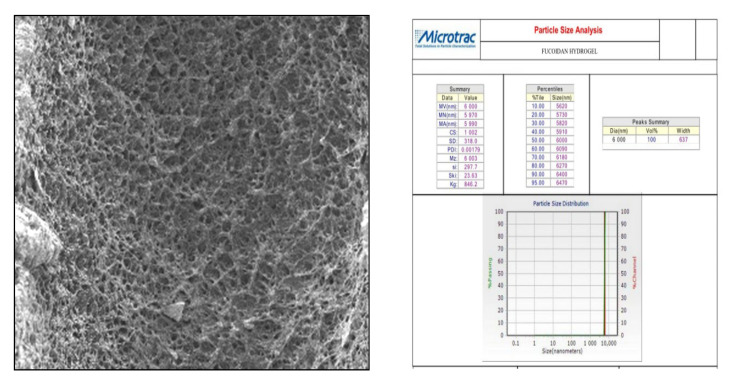
Scanning electron microscopic analysis of fucoidan containing chitosan hydrogel depicting particle size.

**Figure 2:**
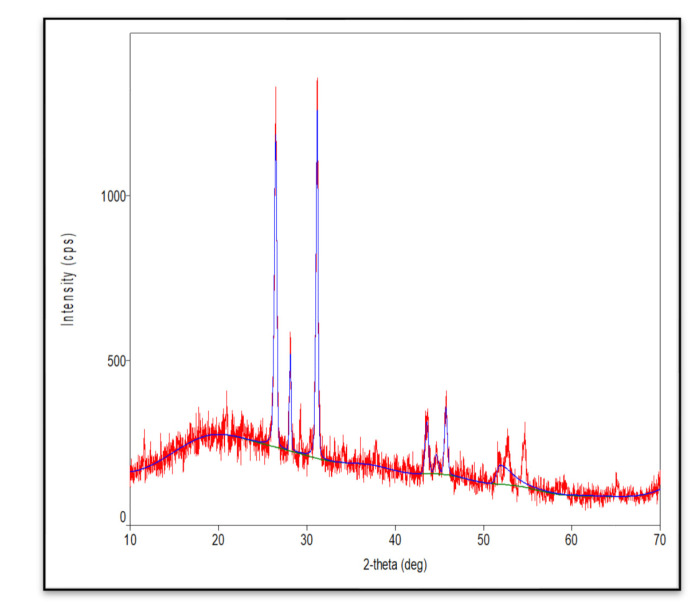
X-ray diffraction analysis of fucoidan containing chitosan hydrogel.

**Figure 3:**
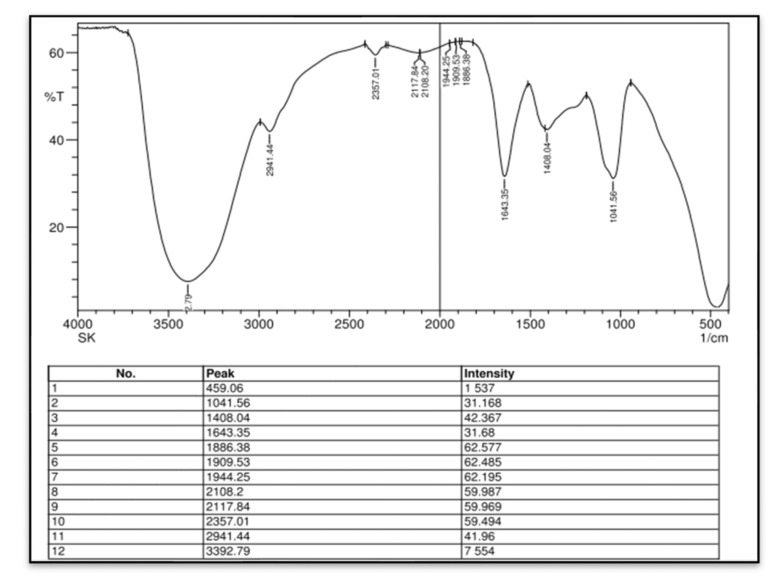
Fourier Transformed Infrared Spectroscopy (FTIR) analysis of fucoidan containing chitosan hydrogel.

In this study, we attempted to develop and compare the effectiveness of a thermosensitive injectable fucoidan-chitosan hydrogel with concentrated growth factor in the regeneration of periodontal intra-bony defects. The results of development and characterization have been published prior [16]; however, this constitutes the complete research on the efficacy and effectiveness of the developed hydrogel. The hydrogel was prepared using the sol-gel method and its physical, chemical, and biological properties stringently scrutinized to assess its efficacy in inducing bone formation. Fucoidan containing chitosan hydrogel was light brown in color, uniformly dispersed with no flakes seen. Rheological results showed the viscosity of the gel as 290.3 ± 0.56 Cps and the gelation time to be 1 to 2 min. The temperature at which the preparation transformed to gel was taken as gelation temperature. The gelation temperature was found to be 32 ± 2 °C. Figure 1 depicting the scanning electron microscopy showed porous structure, indicating micro- and macro-sized pores that were uniform, interconnected, and with good solubility. The particle size as measured using scanning electron microscopy was found to be 6000 nm, with a width of 637 nm, thus, rendering it suitable for nutrient transport, successful cell adhesion and penetration to achieve regeneration. The X-ray diffraction analysis (Figure 2) indicated the crystalline nature of the polymer with peaks at 28 °Ѳ to 30 °Ѳ. FTIR Spectra (Figure 3) showed 1029- (C-O) stretching, 1575 NH bending to amine, 2855 (C-H) stretching and 3433OH-NH- stretch [16].

**Figure 4:**
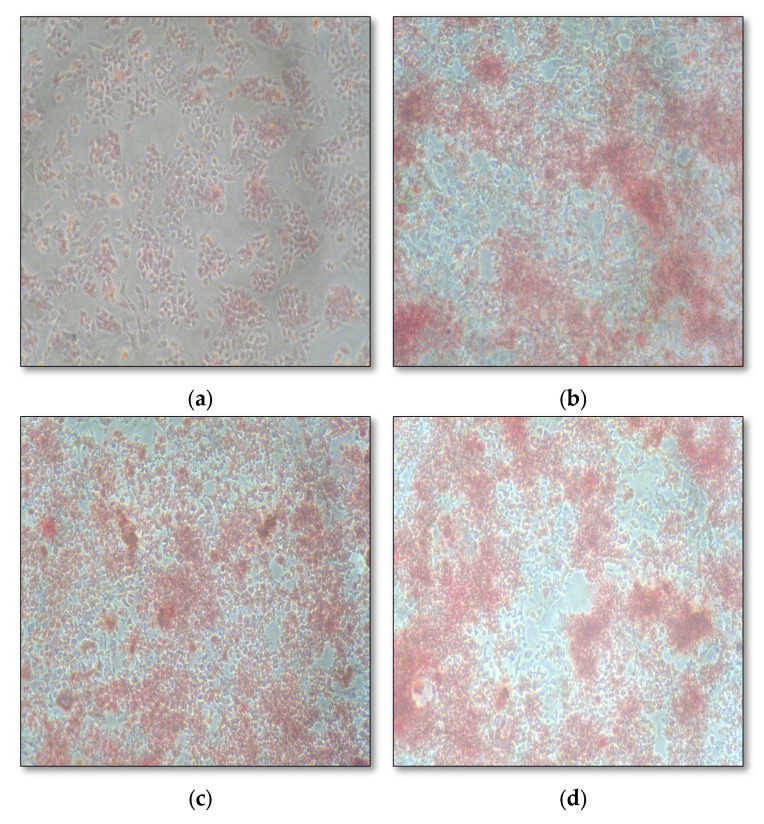
Mineralization assay of fucoidan containing chitosan hydrogel: (**a**) control; (**b**) 1.5% fucoidan; (**c**) 0.5% chitosan; (**d**) 1% fucoidan and 0.25% chitosan.

Biological characterization with MG-63 osteoblasts cell line (Table 1) showed good biocompatibility, biodegradability, slow and sustained drug release, increased cell proliferation, and enhanced alkaline phosphatase secretion (Table 2), with greater mineralization properties (Figure 4). Chitosan, when combined with other cells and synthetic polymers, has been shown to be osteo-promotive and osteo-inductive. Thus, in combination with fucoidan, it takes on an osteogenic role [14,16,17,18]. Histologically, this hydrogel has proven to be an accelerator of bone regeneration in rat tibia [19].

**Figure 5:**
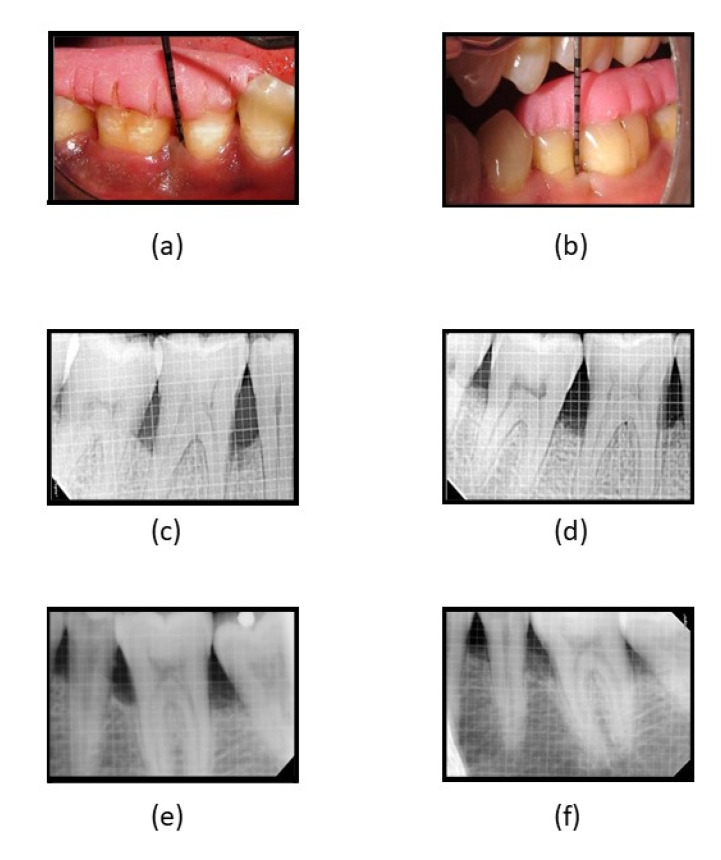
(**a**) Reduction in PPD at 3 months in fucoidan-chitosan group; (**b**) reduction in PPD at 6 months in fucoidan-chitosan group; (**c**) RVG image of intra-bony defect at baseline in fucoidan-chitosan group; (**d**) RVG image of improvement in intra-bony defect at 9 months in fucoidan-chitosan group; (**e**) RVG image of intra-bony defect at baseline in CGF group; (**f**) RVG image of intra-bony defect at 9 months in CGF group.

Clinically, in its osteogenic effectiveness in periodontal intra-bony defects management, the mean age of the participants recruited was 43.75 (5.638) years, with a higher percentage of females (60%, *n* = 24) (Table 3). Statistically significantly lower plaque index scores were observed in both groups at 6 and 9 months as compared to baseline (*p* < 0.05), while significantly lower scores in all other clinical parameters were observed at all three-time intervals when compared to baseline in both groups. Intergroup analyses showed significantly lower scores were observed in the GCF group at 3 and 6 months, while both groups were comparable at 9 months (Table 4). A statistically significant reduction was observed in radiographic parameters at 9 months in both groups (*p* < 0.05). A higher defect fill was seen in the fucoidan-chitosan group at 9 months, with lower CEJ-base measurement (Table 5). The clinical and radiographic improvement is shown in Figure 5.

Literature evidence demonstrates the efficacy of chitosan for regenerating periodontal structures and concludes the superior potential of natural polymers combined with bio-composites, growth factors, gene-activated matrix, and signaling molecules in increasing bioactivity, thereby enhancing bone regeneration [20]. As chitosan’s excellent biocompatibility and degradation are coupled with low bioactivity, a combination with fucoidan was experimented with and showed promising results in treating periodontal intra-bony defects. To the best of our knowledge, this is the first study on fucoidan-chitosan hydrogel for periodontal intra-bony defect correction; no studies showing the effectiveness of fucoidan natural-based polymer for periodontal bone regeneration in humans are available as research in this domain which is still at the level of Phase I trials. 

However, there are three reported studies on the effectiveness of chitosan gel in treating intra-bony defects. Boynuegri D, et al., 2009 [21] reported a pocket depth reduction of 2.60 ± 0.17 mm, gain in the attachment of 1.80 ± 0.12 mm, and intra-bony defect depth was 1.40 ± 0.08 mm at the end of 6 months with 1% chitosan gel with allograft, while Babrawala I, et al., 2019 [22] reported that the use of 15% chitosan gel along with bovine bone graft (Bio-oss, Wolhusen, Switzerland) for three walled defects compared with open flap debridement as a control measure exhibited a significant PPD reduction to 2.90 ± 0.56 mm, CAL gain to 3.10 ± 0.67 mm, reduction in IBD depth to 0.80 ± 0.42 mm, and defect resolution of 78.32 ± 5.80% at 9 months. The effectiveness of chitosan nano hydrogel as a bone regenerative material showed a mean reduction in PPD and CAL in the chitosan group when compared with allograft as reported by Meenakshi SS, et al., 2021 [23]. The results we obtained for the fucoidan-chitosan combination have only strengthened the evidence upholding its regenerative potential. This positive effect can be attributed to the ability of fucoidan in inducing osteogenic differentiation in human-derived stem cells, increasing ALP activity, calcium accumulation, increasing osteocalcin, osteopontin, BMP (Bone Morphogenetic Protein) and RUNX2 (RUNX Family Transcription Factor 2). Fucoidan is known to increase phosphorylation (e (PI3K) isoforms, p110α and p110γ isoforms), and increase expression of β-catenin while chitosan structurally resembles the glycosaminoglycan hyaluronic acid which is found in extracellular matrices of many tissues [12,15].

This study is not without limitations. While the sample size used in this study was within the range adopted by most clinical regenerative studies in humans, scope exists to reproduce this protocol with a larger sample size to revalidate the present study findings. The limited sample size limits the external validity or generalizability. Another limiting factor was ethical restraints, due to which surgical re-entry and histologic investigation could not be performed to assess the nature of the regenerated bone.

## 3. Conclusions

Fucoidan containing chitosan hydrogel is biocompatible with improved bioactivity. The addition of fucoidan to chitosan increased mineralization as well as increased ALP activity indicating the potential role in tissue engineering. Fucoidan containing chitosan hydrogel clinically exhibited statistically significantly lower PPD values as compared to the CGF group at the end of 3 and 6 months. The mean reduction in clinical attachment level with the combination was significantly higher than CGF, with an increased bone defect fill in the combination at 9 months, thus proving the efficacy of fucoidan–chitosan hydrogel as a promising material for tissue engineering applications. 

## 4. Materials and Methods

### 4.1. Preparation of Fucoidan Containing Chitosan Hydrogel

Chitosan (Everest Biotech Pharma, Bangalore, India) was subjected to purification by dissolution-precipitation, dialysis, and deacetylation up to an 85% degree to produce gels at 37 °C following simple neutralization with sodium hydroxide, in the following three steps: (a) Deprotonation: On exposure to sodium hydroxide, the hydroxide ions (OH^−^) react with the amino groups (-NH_2_) present in chitosan to remove protons (H^+^) from the amino groups, resulting in their deprotonation and conversion into amino groups (-NH). (b) Gel Formation: The deprotonation of chitosan reduces the electrostatic repulsion between chitosan chains, thus bringing the chitosan chains closer together to form a gel-like structure. The process is primarily driven by the formation of intermolecular hydrogen bonds and entanglement of chitosan chains. (c) Cross-Linking: This gel formation of chitosan by NaOH is further enhanced through the addition of cross-linking agents that promote the formation of covalent bonds between chitosan chains, increasing the stability and strength of the resulting gel. The gel formation was slow with a weak increase in viscosity to produce a stable formulation for a medical application [24,25].

Fucoidan from brown seaweed species, *Sargassum wightii*, was collected, dried overnight in an oven, milled, and strained. A total of 1 g/mg of seaweed was mixed with 10 mL solvent, stored for 2 days, and centrifuged at 10,000 rpm for 15 min of which 20 mg powder was treated with ethanol solvent, stirred for 12 h, and centrifuged for 20 min [26]. To prepare the hydrogel, gelatin was dissolved homogeneously in 100 mL of water by stirring for 1 h, to which 100 mg of fucoidan was added with the help of a dropper while the stirring continued for another hour to dissolve the entire polymer in gelatin. Chitosan was then added to the mixture solution and was continuously stirred for 2 h until it attained homogeneity. A total of 0.1% methyl paraben was added to the gel as a preservative and the hydrogel was sterilized by autoclaving (121 c for 15 min).

### 4.2. Characterization of Fucoidan Containing Chitosan Hydrogel

Rheological studies—The rheological behavior of the gel was measured at 35 ± 2 °C using Brookfield AMETEK (Middleborough, MA, USA) viscometer with multiple trials conducted to choose the appropriate spindle for analyzing the gel. Based on the % Torque value, SC4-28 spindle with temperature control unit was used and the viscosity of the gel was evaluated at 100 rpm with 85% torque value. 

Gelling time—The time taken for sol to gel transformation was measured by test tube inversion method where 1 mL of the liquid formulation was collected in a cleaned glass test tube and placed in a temperature-controlled water bath at 37 ± 2 °C. The time taken for gelling was determined.

Gelation temperature—Thermosensitive chitosan gel was placed in a test tube, containing 0.5 mL phosphate buffer solution with pH at 6.8 and was placed in a cold-water bath maintained to 20  ± 1 °C. The temperature of the water bath was then gradually raised to 40 °C, with the increment of 2 °C for every 5 min. The change in consistency of the formulation with the increment of temperature was recorded. The temperature at which the preparation transformed to gel was taken as gelation temperature.

Scanning Electron Microscopy (SEM)—SEM assessed surface morphology and pore structure of the developed hydrogel. Using a scalpel, a section of hydrogel was segmented and mounted on aluminum stubs. The samples were cut precisely into small pieces, fixed on carbon tapes, dried using vacuum, and gold-coated, following which, these were subjected to SEM analysis.

X-ray Diffraction Analysis—The crystalline nature was studied by grounding the samples to powder form in liquid nitrogen and analyzed by XRD for two angles between 5° to 80° at a speed of 2° per minute using an analytical XPERT PRO powder diffractometer operating at a voltage of 40 kV (Cu K radiation).

Fourier Transform Infrared Spectroscopy—FTIR analyzed the intermolecular chemical interactions between the various functional groups within the components present in the fucoidan containing chitosan hydrogel.

Cytotoxicity studies—Concentrations of 0.5%, 1%, and 1.5% were prepared using Dulbecco’s Modified Eagle Medium (DMEM) culture media with other chemicals to culture cells in an atmosphere of 5% CO_2_ at 37 °C until confluence. Standardization was performed using 2%, 3%, and 4% concentrations of DMEM plain media. Cells were separated using a dissociation solution, and the viability of the cells was checked, with cells (50,000 cell/wall) suspended in a 96-well plate. Each well was filled with 100 L of cells and incubated for 24 h at 37 °C in 5% CO_2_ incubator. After 24 h, the supernatant layer was removed and washed, followed by testing of various concentrations of drug using the same procedure. Formation of formazan was measured with a microplate reader and the amount of drug needed to inhibit cell growth was calculated from each cell line.

Osteoblast Re-mineralization Assay—Alizarin Red Sulphonic acid stain was used to identify calcium deposits, indicating the formation of bone. Culture 1 × 10^6^ cells in four P35 dishes containing 2 mL of complete DMEM media was used, in which, following 24 h incubation, cells were treated with various concentrations of fucoidan and chitosan such as 1.5% fucoidan, 0.5% chitosan, and 1% fucoidan + 0.25% chitosan. The control well included 2 mL of serum-free DMEM media and all the four P35 dishes incubated for the next 24 h. Every third day, media was changed with test samples for 21 days, following which, the cell monolayer was washed with 1X PBS without disrupting the cell monolayer. Carefully aspirated, the buffer was discarded, and the cells were fixed using 10% formalin, incubated for 30 min, washed with distilled water, and treated with Alizarin Red S stain to cover the cell monolayer and incubate the cells in the dark for 45 min. On repeating the procedure four times, the samples were subject to microscopic analysis to appreciate the calcium deposits. 

Alkaline phosphatase activity—MG63 monolayer cell culture was dissociated and adjusted 1 × 10^5^ cells/mL using 10% Foetal Bovine Serum in a 96-well microtiter plate where each of the wells was immersed with 100 L of cell suspension and incubated for 24 h. The partial monolayer formed was discarded and washed. Different test concentrations (100 L) of the samples were added in well plates and incubated at 37 °C for 24 h in a 5% CO_2_ atmosphere. After incubation, to 20 µL of test solutions, 1000 µL working reagent was added. OD was taken at 405 nm every 1 min for 3–4 times and the ALP activity was calculated.

### 4.3. Comparative Evaluation of the Effectiveness of Fucoidan Containing Chitosan Hydrogel against Concentrated Growth Factor (CGF) in Periodontal Intra-bony Defects

A randomized controlled trial clinical trial, following CONSORT guidelines, was conducted among subjects visiting the outpatient services in the Department of Periodontics, KLE Society’s Institute of Dental Sciences, Bangalore, India. The sample size was estimated considering a small effect size (0.2) for the two-tailed hypothesis, keeping the power at 80% and the margin of the alpha error at 5% using GPower software v. 3.1.9.2. (Heinrich-Heine University) [27]. A total of 40 subjects with 20 in each study group were recruited using the convenience sampling method. 

The study was followed up for nine months to evaluate the effectiveness of fucoidan-chitosan nano hydrogel and concentrated growth factor as a bone regenerative material in periodontal intra-bony defects. Before commencing, clearance for the study was obtained from the institutional ethics committee and written informed consent was obtained from every participant. Subjects aged between 25 to 50 years, without the presence of systemic diseases, non-smokers with chronic periodontitis, and a pocket depth of 5–7 mm [28] accompanied by two wall intra-bony defects and less than <25 defect angle were recruited. Subjects who have undertaken the treatment within a year, pregnant women, and having mobile furcation-involved teeth were excluded from the study.

Preparation of concentrated growth factor: CGF was prepared by collecting 10 mL venous blood which was centrifuged for 4 min at 2700 rpm. After centrifugation, the layer with growth factor was carried with the instrument and adapted to the membrane [29]. The gels were loaded in 2 mL short needle syringes for ease of placement in the defect site during periodontal surgery. The surgical operator and the subjects were blinded to the study groups and the test products.

Study Method—Before the commencement of the study, personalized acrylic stents were designed for each patient to fit the selected teeth. At the defect site on the stent, vertical grooves were made to guide the probe penetration in the plane. Detailed history and periodontal examination were performed for all the study participants, following which phase I therapy consisting of scaling, root planning, and oral hygiene instructions was completed. 

Clinical measurements recorded by a single examiner included Plaque Index (PI) by Silness and Loe (1964) [30], Gingival Index (GI) by Loe and Silness (1963) [31], Periodontal Probing Depth (PPD), and Clinical attachment level (CAL). The former two indices were evaluated as 0–3, based on the range of plaque and gingivitis present. An overall score was then estimated for each participant. The latter two are manifestations of periodontitis where PPD is measured from the gingival margin, that usually changes across time to the base of the pocket, while CAL relies on a fixed reference point like the cementoenamel junction, until the base of the pocket. Both the measurements have been recorded in mm using a graduated periodontal probe at six sites around each tooth, namely Mesiobuccal, Midbuccal, Distobuccal, Mesiolingual, Midlingual, and Distolingual points. The highest value recorded at any of these sites is recorded as the PPD/CAL value for the tooth. Mean values are estimated for each subject at baseline, 3 months, 6 months, 9 months, and 12 months follow-up. Radiographic assessment was carried out at baseline and 9 months follow-up using radiovisiography (RVG-Suni Medical Imaging, Apteryx Inc., Acron, OH, USA) to assess the intra-bony depth, which was measured as the vertical distance from the crest of the alveolar bone to the base of the defect. All radiographs were assessed independently by one blinded examiner using the computer-aided program Digimizer for analysis. 

Participants were then randomized in 1:1 allocation randomization sequence using a computer-generated table of random numbers into Group 1 receiving the fucoidan-chitosan hydrogel and Group 2 receiving concentrated growth factor. The respective surgical procedure was performed and assessed for the outcome. All the clinical and radiographic measurements and surgeries were performed by one examiner who was blinded to the study groups. Patients were blinded for allocation to a particular group and treatment. The study design and participant flow are provided in the Supplemental Materials (Figure S1).

**Figure 6:**
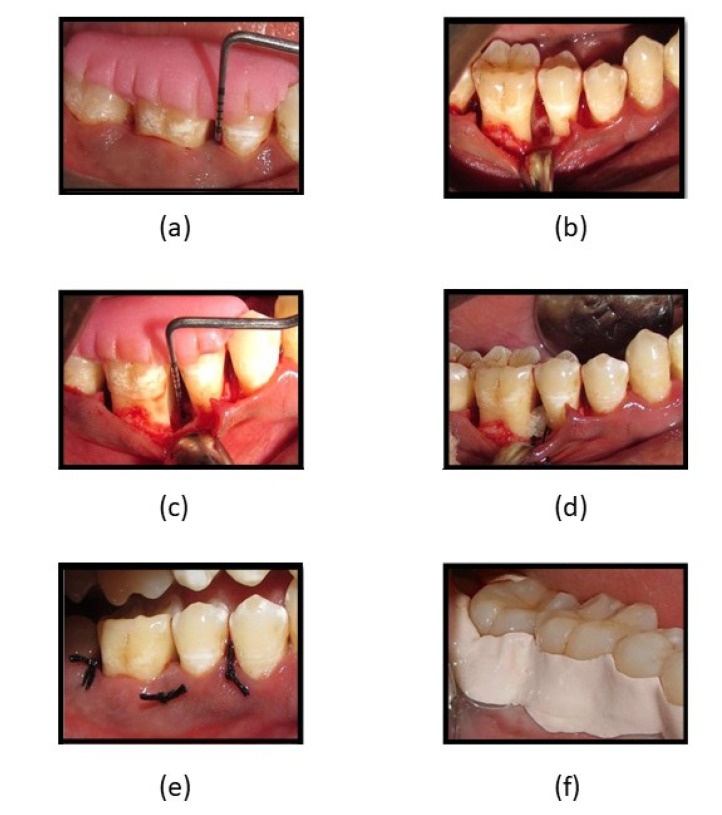
(**a**) PPD and CAL measuring 8-mm; (**b**) flap reflection, debridement, and surgical procedure of intra-bony defect; (**c**) intra-surgical measurement of intra-bony defect CEJ to depth of the bony defect; (**d**) fucoidan-chitosan gel placed into intra-bony site; (**e**) sutures placed and intra-bony defect covered by flap; (**f**) periodontal pack placed.

Surgical procedure (Figure 6)—Before the surgical procedure, the defect site was anesthetized by administering 2% lignocaine and an epinephrine concentration of 1:80,000, following which sulcular incisions were made on the buccal and lingual aspects of the involved sites and full-thickness mucoperiosteal flaps were raised. Area-specific curettes and ultrasonic scalers were used for thorough debridement, after which, the defect site in participants allocated to Group 1 was filled with fucoidan containing chitosan hydrogel as a bone regenerative material, and Group 2 had CGF for bone regeneration. Simple interrupted sutures were placed to achieve primary wound closure followed by the placement of periodontal dressing.

Postoperative wound management—Immediately after surgery, 500 mg of amoxicillin every 6 h for 5 days, 400 mg of Ibuprofen every 8 h, and 0.2% chlorhexidine digluconate mouthwash twice daily for 4 weeks were prescribed to all the participants. They were re-evaluated for pain, sensitivity, and discomfort, and sutures were removed after 7 days. 

Statistical Analysis—Descriptive statistics were computed for all study parameters. Data were normally distributed as assessed by the Shapiro–Wilk test. The difference in the mean measurements of hard and soft tissue parameters between the two groups at all time points was assessed using the independent samples *t*-test, while intragroup comparisons across the time intervals were assessed using paired *t*-test and repeated measures ANOVA, followed by Bonferroni post hoc analysis. The difference in the values was significant at a *p*-value of less than 0.05.

## Figures and Tables

**Table 1:** Results of biocompatibility of fucoidan containing chitosan hydrogel.

Formulations	Fucoidan %	Chitosan %	OD-590 nm	Cytotoxicity %
1	4.5	2	0.419	54.06
2	3	1	0.429	52.96
3	1.5	0.5	0.659	27.74
4	1	0.25	0.832	8.77
5	0.5	0.5	0.751	17.65

**Table 2:** ALP activity of fucoidan containing chitosan hydrogel.

Sample	Concentration µg/mL	AT 1 MIN	AT 3 MIN	ALP Activity
		Absorbance @ 405 nm	Absorbance @ 405 nm	
Control	0	0.324	0.325	0.91
Hydrogel	0.5%	0.806	0.859	48.05
Reso Sod	3.125	0.291	0.298	6.62
	6.25	0.325	0.338	11.80
	12.5	0.474	0.498	21.55
	25	0.532	0.560	25.02
	50	0.612	0.648	32.37
	100	0.785	0.835	45.33

**Table 3:** Age and gender distribution.

Age Group	*n* = 40	%
31–40 years	12	30
41–50 years	23	57.5
>50 years	5	12.5
Age	Mean	SD
Mean Age (years)	43.75	5.638
Gender	*n* = 40	%
Males	16	40
Females	24	60

**Table 4:** Comparison of clinical parameters between study groups.

Clinical Parameters	Fucoidan-Chitosan Hydrogel		CGF		*t*-Statistic	*p*-Value ^$^ Group 1 vs. 2
	Mean	SD	Mean	SD		
N	20		20			
Plaque Index						
Baseline	1.515	0.2641	1.465	0.2681		
3 months	1.265	0.2581	1.445	0.3379	2.192	0.037 ^$^
*p*-value *	<0.001 *		1.000			
6 months	1.140	0.2479	0.930	0.1081	−2.297	0.027^$^
*p*-value *	<0.001 *		<0.001 *			
9 months	0.945	0.1234	0.940	0.1231	0.665	0.510
*p*-value *	<0.001 *		<0.001 *			
Gingival Index						
Baseline	1.550	0.3426	1.620	0.3270		
3 months	1.375	0.3596	1.220	0.2726	−2.239	0.036^$^
*p*-value *	<0.001 *		0.004 *			
6 months	1.320	0.3238	1.370	0.3045	−0.483	0.632
*p*-value *	<0.001 *		<0.001 *			
9 months	1.010	0.1119	1.055	0.1605	−0.291	0.773
*p*-value *	<0.001 *		<0.001 *			
Periodontal Probing Depth						
Baseline	7.55	0.887	7.80	0.894		
3 months	5.50	0.946	6.80	0.523	4.098	<0.001 ^$^
*p*-value *	<0.001 *		<0.001 *			
6 months	5.50	0.946	6.35	0.489	2.214	0.033 ^$^
*p*-value *	<0.001 *		<0.001 *			
9 months	4.50	0.513	5.05	0.224	0.993	0.327
*p*-value *	<0.001 *		<0.001 *			
Clinical Attachment Level						
Baseline	8.40	0.598	8.45	0.510		
3 months	7.30	1.031	6.80	0.523	−2.377	0.023 ^$^
*p*-value *	<0.001 *		<0.001 *			
6 months	6.85	1.461	6.35	1.387	0.948	0.234
*p*-value *	0.001 *		<0.001 *			
9 months	5.15	0.489	5.15	0.366	−0.261	0.796
*p*-value *	<0.001 *		<0.001 *			

* Indicates intragroup comparison (vs. baseline) assessed using repeated measures ANOVA with Bonferroni’s post hoc test. ^$^ Indicates intergroup comparison (vs. CGF) assessed using independent sample *t*-test.

**Table 5:** Comparison of radiographic parameters between study groups.

Clinical Parameters	Fucoidan-Chitosan		CGF		*t* Statistic	*p*-Value ^$^ Group 1 vs. 2	
	Mean	SD	Mean	SD			
N	20		20				
	Cementoenamel Junction to Base						
Baseline	6.70	1.302	6.60	1.353			
9 months	4.95	0.759	6.10	0.718			
*p*-value *	<0.001 *		0.008 *		3.817	<0.001 ^$^	
	Cementoenamel Junction to Bone Level						
Baseline	3.55	0.510	5.85	0.745			
9 months	2.90	0.447	4.90	0.553			
*p*-value *	<0.001 *		<0.001 *		−1.485	0.146	
	Intra-bony Defects						
Baseline	3.35	0.489	3.45	0.510			
9 months	2.10	0.308	2.05	0.224			
*p*-value *	<0.001 *		<0.001 *		−0.900	0.374	
	Defect Fill						
9 months	1.20	0.410	0.20	0.410	7.706	<0.001 ^$^	

* Indicates intragroup comparison (vs. baseline) assessed using paired sample *t*-test. ^$^ Indicates intergroup comparison (vs. CGF) assessed using independent sample *t*-test.

## Data Availability

The data presented in this study are available on request from the corresponding author. The data are not publicly available due to ethical reasons and confidentiality.

## Supplementary Materials

The following supporting information can be downloaded at: https://www.mdpi.com/article/10.3390/gels9070573/s1, Figure S1: Framework of participant flow.
