# Supplementary figures and images for: Evaluation of Osteogenic Potential of Fucoidan Containing Chitosan Hydrogel in the Treatment of Periodontal Intra-Bony Defects—A Randomized Clinical Trial
Source: Gels. 2023 Jul 13;9(7):573. doi: 10.3390/gels9070573 (PMC10379738; doi:10.3390/gels9070573)

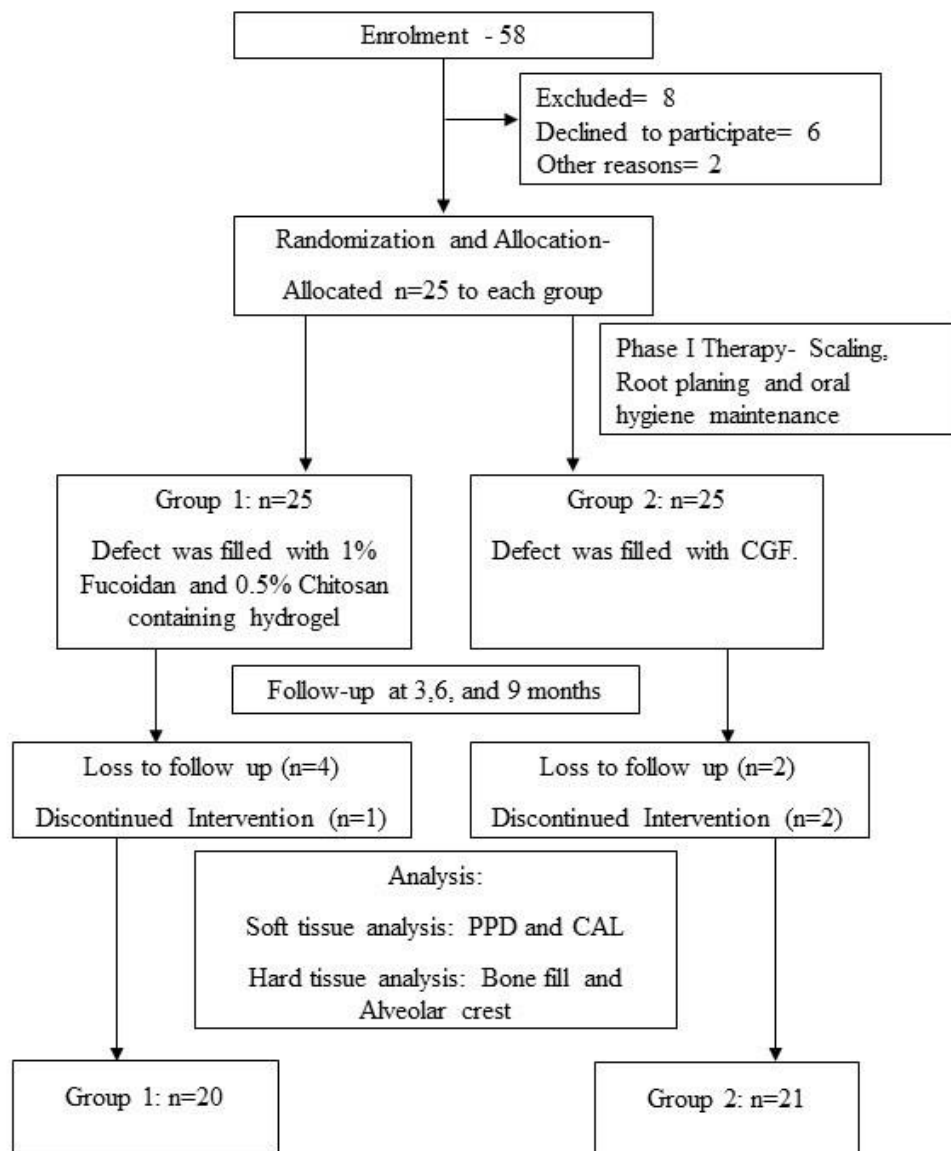

**Figure S1.** Framework of participant flow

Supplement: Supplementary file 1 [file gels-09-00573-s001.zip › gels-2448995-supplementary.pdf]
